# Urgency for Digital Technologies to Support Caregivers. Comment on “Telehealth-Based Psychoeducation for Caregivers: The Family Intervention in Recent-Onset Schizophrenia Treatment Study”

**DOI:** 10.2196/40147

**Published:** 2022-06-30

**Authors:** Jens Peter Eckardt

**Affiliations:** 1 Bedre Psykiatri Research Unit Copenhagen Denmark

**Keywords:** schizophrenia, family psychoeducation, caregiver burden, recent-onset schizophrenia, telehealth

I read with great interest the article “Telehealth-Based Psychoeducation for Caregivers: The Family Intervention in Recent-Onset Schizophrenia Treatment Study” by Mueser et al [1].

First, I would like to congratulate the authors for raising awareness of telehealth-based psychoeducation for informal caregivers of people with mental illness. The results of their study present important understandings and hope for continued progress in the web-based delivery of valuable family interventions within the field of psychiatry. Although this study did not show the family psychoeducation intervention to be advantageous at the level of exposure reached, explorations of future caregiver-focused, telehealth-based interventions in mental health are still needed.

As we look toward future development in working with families and informal caregivers of people with mental illness, digital innovations, applications, and advancements in web-based interventions [2] suggest the importance of supporting more research, strategies, and exploration of sufficient and continuous caregiver engagement and involvement. Such approaches are critically desired and have the potential to engage more informal caregivers and families in a cost- and time-effective manner, offer prospects for online social networking, and provide more elasticity in informal caregiver decisions of where, when, and how they choose to be involved with the intervention or application. Additionally, such innovations could make mental health resource allocation less challenging.

Compared to patient groups, telehealth-based interventions with family groups have been limited [3] despite the extensive existing quality research on family engagement and the effectiveness of family interventions in mental health services [4]. The findings of Mueser et al [1] imply that digital technology and engagement strategies can be used successfully to meet the needs of informal caregivers and families for psychoeducation and network support throughout a considerable geographical area and, therefore, have the potential to fill a significant gap in delivering high-quality interventions and support to informal caregivers of people with mental illness [5].

In light of accelerating digital interventions within the field of psychiatry, I call on the research community to also draw more attention to the potential of telehealth-based psychoeducation for informal caregivers of people with mental illness. With the expected growth in the caregiver population coupled with significant caregiver needs and demands for help and support, notwithstanding those who are unable to join or have no access to these mental health services or best practices, taking advantage of the future potential of digital technologies and interventions for caregivers is inevitable.
